# Immunotherapy with Tolerogenic Dendritic Cells Alone or in Combination with Rapamycin Does Not Reverse Diabetes in NOD Mice

**DOI:** 10.1155/2013/346987

**Published:** 2013-03-11

**Authors:** Irma Pujol-Autonell, Rosa M. Ampudia, Pau Monge, Anna M. Lucas, Jorge Carrascal, Joan Verdaguer, Marta Vives-Pi

**Affiliations:** ^1^Laboratory of Immunobiology for Research and Diagnosis (LIRAD-BST), Germans Trias i Pujol Research Institute, Autonomous University of Barcelona, 08916 Badalona, Spain; ^2^Department of Endocrinology, Germans Trias i Pujol University Hospital, Autonomous University of Barcelona, 08916 Badalona, Spain; ^3^Immunology Unit, Department of Ciencies Basiques Mediques, Faculty of Medicine, University of Lleida & IRBLleida, 25198 Lleida, Spain

## Abstract

Type 1 diabetes is a metabolic disease caused by autoimmunity towards **β**-cells. Different strategies have been developed to restore **β**-cell function and to reestablish immune tolerance to prevent and cure the disease. Currently, there is no effective treatment strategy to restore endogenous insulin secretion in patients with type 1 diabetes. This study aims to restore insulin secretion in diabetic mice with experimental antigen-specific immunotherapy alone or in combination with rapamycin, a compound well known for its immunomodulatory effect. Nonobese diabetic (NOD) mice develop spontaneous type 1 diabetes after 12 weeks of age. Autologous tolerogenic dendritic cells—consisting in dendritic cells pulsed with islet apoptotic cells—were administered to diabetic NOD mice alone or in combination with rapamycin. The ability of this therapy to revert type 1 diabetes was determined by assessing the insulitis score and by measuring both blood glucose levels and C-peptide concentration. Our findings indicate that tolerogenic dendritic cells alone or in combination with rapamycin do not ameliorate diabetes in NOD mice. These results suggest that alternative strategies may be considered for the cure of type 1 diabetes.

## 1. Introduction

Type 1 diabetes (T1D) results from the autoimmune destruction of insulin-producing *β*-cells in the pancreatic islets of Langerhans [1]. The prevalence of this disease, its complications, and the lack of effective curative and preventive strategies call for a significant effort to find means to restore the tolerance to *β*-cells as the best way to control this disease. Recently, we reported a new experimental immunotherapy protocol based on the use of dendritic cells (DCs) loaded with islet apoptotic cells. This protocol clearly reduces T1D incidence and insulitis in non-obese diabetic mice (NOD) [2]. The NOD mouse strain is an excellent model for autoimmune diabetes.

A limited number of treatments were demonstrated to revert established diabetes in NOD mice [3, 4]. Given the preventive effectiveness of DCs loaded with islet apoptotic cells, we tested whether this immunotherapy is able to reverse diabetes. To improve this treatment, we also combined tolerogenic DCs with rapamycin, an immunosuppressant that inhibits the response to IL-2 and thereby blocks the activation of T and B lymphocytes.

Rapamycin, a noncalcineurin-based inhibitor, was used to prevent acute graft rejection following allogeneic transplantation [5] and helps to expand T regs [6]. Therefore, we hypothesized that the administration of rapamycin prior to immunotherapy might help to reduce insulitis in diabetic mice, thus facilitating the subsequent action of antigen specific immunotherapy. Our findings indicate that tolerogenic DCs do not ameliorate diabetes in NOD mice in terms of blood glucose levels, endogenous insulin secretions, or insulitis score. Surprisingly, therapy with rapamycin does not improve the metabolic conditions in mice treated with tolerogenic DCs.

## 2. Materials and Methods

### 2.1. Animals

This study was carried out in strict accordance with the recommendations in the Guide for the Care and Use of Laboratory Animals of the Generalitat de Catalunya, Catalan Government. The protocol was approved by the Committee on the Ethics of Animal Experiments of the Germans Trias i Pujol Research Institute(Permit number: DAAM 5157). Wild type NOD mice were obtained from our colony established with mice from the Jackson Laboratory (Bar Harbor, ME, USA). Mice were kept under specific pathogen-free conditions and monitored daily for diabetes assessment. Mice with glycosuria were confirmed diabetic when either successive blood glucose levels were higher than 200 mg/dL, or when a measure was higher than 360 mg/dL. Mice were treated with daily s.c. insulin (1U, Insulatard FlexPen, Novo-Nordisk, Bagsvaerd, Denmark). Diabetic NOD mice (12- to 17-weeks old) were divided into four groups: (1) sham (nontreated control group), (2) Rapa (treated with rapamycin), (3) NITApo-DCs (treated with tolerogenic DCs), and (4) Rapa + NITApo-DCs (treated with rapamycin and tolerogenic DCs). A total of 3–6 animals were included in each group. Sham group animals were injected with 0.15 mL physiological saline solution. Mice were monitored for urine glucose using Glucocard strips (Menarini, Barcelona, Spain) daily for 30 days. Blood glucose levels were monitored weekly (AccuCheck, Roche Diagnostics, Indianapolis, IN) after fasting for 2 hours. Blood was collected for C-peptide assay at the end of the follow-up.

### 2.2. Treatment with Tolerogenic Dendritic Cells

DCs were propagated from bone marrow progenitors of female wild type NOD mice in a culture medium containing GM-CSF (Prospec, Rehovot, Israel) as previously described [2]. At the same time, *in vitro* cultured NIT-1 cells obtained from the American Type Culture Collection (ATCC, Manassas, VA) and derived from *β*-cells from NOD/Lt mice [7] were submitted to apoptosis by UVB irradiation, as described [2]. Tolerogenic DCs were prepared by coculturing DCs with islet apoptotic bodies in a 3 : 1 ratio for 2 hours. DCs loaded with apoptotic bodies from NIT-1 cells (NITApo-DCs) were separated from unloaded DCs by sorting (FACSAria II, BD Biosciences). NIT-1 cells were prelabelled with CFSE (Molecular Probes, Invitrogen, Carlsbad, CA) to allow the separation of DCs pulsed with apoptotic bodies (CD11c and CFSE positive). Diabetic NOD mice were given a single intraperitoneal dose of 10^6^ NITApo-DCs (NITApo-DCs group) in 150 *μ*L saline solution, seven days after the onset of the disease. 

### 2.3. Treatment with Rapamycin

Treatment with rapamycin (Rapamune, Pfizer Inc, Bedminster, NJ) was orally administered at the clinical onset of diabetes. Rapamycin was diluted in water. During the first week, rapamycin was administered once daily at a dose of 2.5 mg/kg by gavage (gauge 20G, Fine Science Tools, Foster City, CA). After the administration of immunotherapy at day 7, rapamycin was given in alternate days until the end of the follow-up (30 days) (Rapa + NITApo-DCs group). A control group receiving rapamycin, but not immunotherapy with DCs, was also included (Rapa group) in the study.

### 2.4. Insulitis Score

The degree of islet infiltration by leukocytes—insulitis—was determined at the end of the study. Briefly, the pancreases from all the animals of each group were snap frozen in an isopentane/cold acetone bath. Cryosections of 5 *μ*m were obtained at five nonoverlapping levels, stained with hematoxylin and eosin (H&E), and analyzedby two independent observers who were blinded to the experimental conditions. Each observer assessed a minimum of 40 islets per animal. Insulitis was scored as described elsewhere [2]: 0, no insulitis; 1, peri-insular; 2, mild insulitis (<25% of infiltrated islets); 3, severe insulitis (25–75% of infiltrated islets); 4, destructive insulitis (complete islet infiltration).

### 2.5. Endogenous Insulin Production

The recovery of *β*-cell function was assessed by measuring C-peptide levels in sera at the end of the study, after fasting for 2 hours. The C-peptide concentration (pg/mL) was determined by ELISA (Raybiotech, Norcross, GA). The sensitivity of the assay is 772 pg/mL. Reproducibility Intra-Assay: CV < 10% and Inter-Assay: CV < 15%. This ELISA shows no cross-reactivity with any of the cytokines tested: ghrelin, nesfatin, angiotensin II, NPY, and APC.

### 2.6. Statistical Analysis

The results were expressed as averages of each group ± standard deviation (SD). The comparisons between groups used the unpaired *t*-test for unpaired data after confirming normal distributions. A *P*-value < 0.05 was considered significant.

## 3. Results

### 3.1. Tolerogenic DCs—Alone or in Combination with Rapamycin—Do Not Reverse T1D in NOD Mice

**Figure 1:**
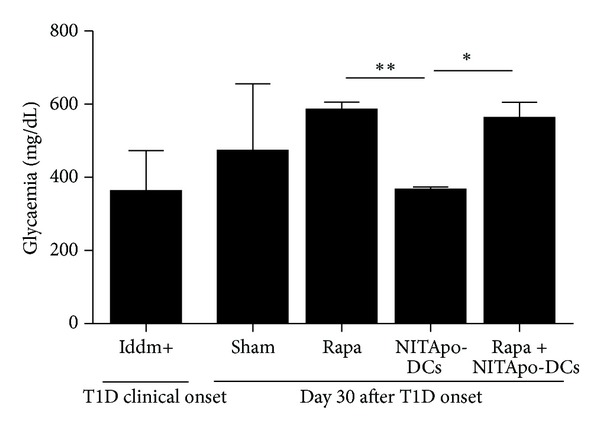
Immunotherapy—alone or in combination with immunosuppressant—does not reverse T1D in NOD mice. Glucose levels in diabetic mice from different groups. White bar corresponds to glycaemia in a group of 8 mice at the onset of the disease. Black bars correspond to different groups at the end of the study (day 30). No significant differences were observed when treated groups were compared to the sham group. Significant differences were found when the NITApo-DCs group was compared with mice treated with rapamycin (Rapa) and with mice treated with tolerogenic DCs and rapamycin (Rapa + NITApo-DCs). Results are means ± SD from 3–6 mice per group. ∗ and ∗∗ mean significant differences, *P* < 0.05 and *P* < 0.01, respectively.

Mice were spontaneously rendered diabetic after 13 weeks of age showing elevated blood glucose concentration (363.5 ± 109.6 mg/dL, mean ± SD). To assess the efficacy of tolerogenic DCs for curing T1D we treated diabetic NOD mice seven days after the onset of the disease. Fasting blood glucose levels were not significantly different between mice treated with NITApo-DCs immunotherapy and the sham group at the end of the study (Figure 1). The administration of rapamycin did not improve glycaemia when compared to the sham group. Moreover, no effect in glucose levels was observed with the combination of both agents, with or without immunosuppression. Among the three treated groups, the lowest glycaemia was achieved by mice treated with tolerogenic DCs, showing significant differences when compared to mice treated with rapamycin alone or in combination with tolerogenic DCs. However, all diabetic NOD mice sustained hyperglycaemia at the end of the study.

### 3.2. Insulitis Is Not Reduced by Tolerogenic DCs in Diabetic NOD Mice

**Figure 2:**
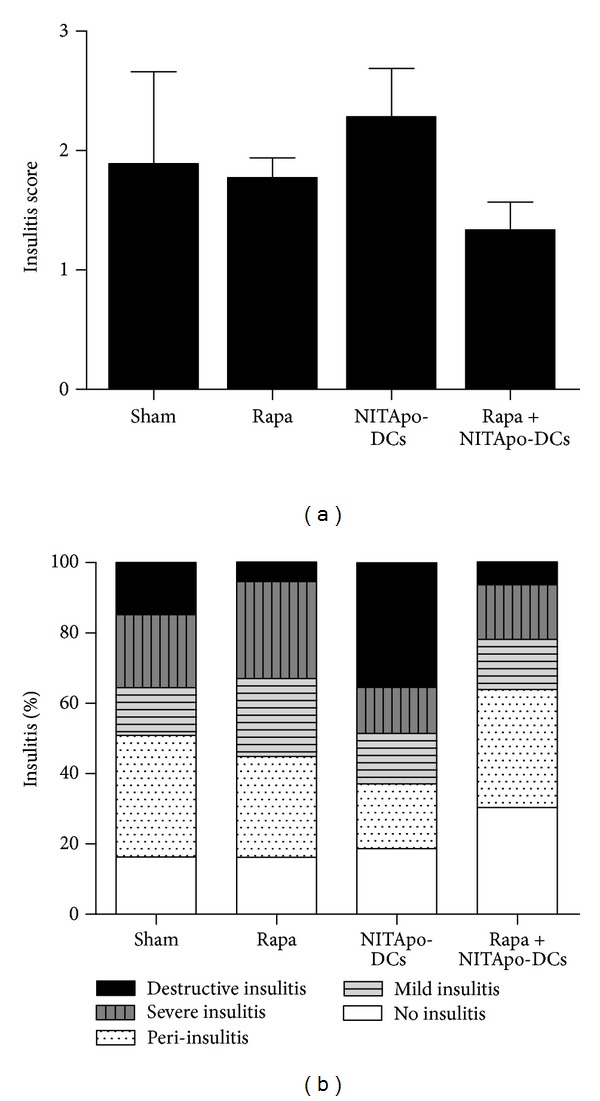
Insulitis is not reduced by immunotherapy and/or rapamycin administration in diabetic NOD mice. Effect of the treatment with tolerogenic DCs and/or an immunosuppressant (rapamycin) on insulitis in NOD mice. (a) Insulitis score for different groups. The pancreas from 3 animals/group was analyzed by two independent observers at the end of the study period (30 weeks). Each observer assessed a minimum of 40 islets per animal. Results are means ± SD. (b) The percentage of classified islets in each of the five infiltration categories in different groups was as follows: 0, no insulitis; 1, peri-insulitis; 2, mild insulitis (<25% of infiltrated islets); 3, severe insulitis (25–75% of infiltrated islets); 4, destructive insulitis (complete islet infiltration).

Insulitis was scored in diabetic mice 30 days after treatment to determine the islet leukocytic infiltration degree. No significant differences were found between the four groups of diabetic mice (Figure 2(a)). The insulitis score in mice treated with tolerogenic DCs-based immunotherapy was similar to that in the sham group. The insulitis score in mice treated with rapamycin in combination of tolerogenic DCs was similar to that in mice treated with rapamycin alone. The percentage of islets classified in each of the five infiltration categories in different groups showed that the severity of insulitis correlates with the percentage of islets with destructive insulitis and severe insulitis (Figure 2(b)). Insulitis-free islets were less than 30% in groups of diabetic mice.

### 3.3. Endogenous Insulin Secretion Ameliorates in Mice Treated with Rapamycin but the Effect Is Counteracted When Combined with Tolerogenic DCs

**Figure 3:**
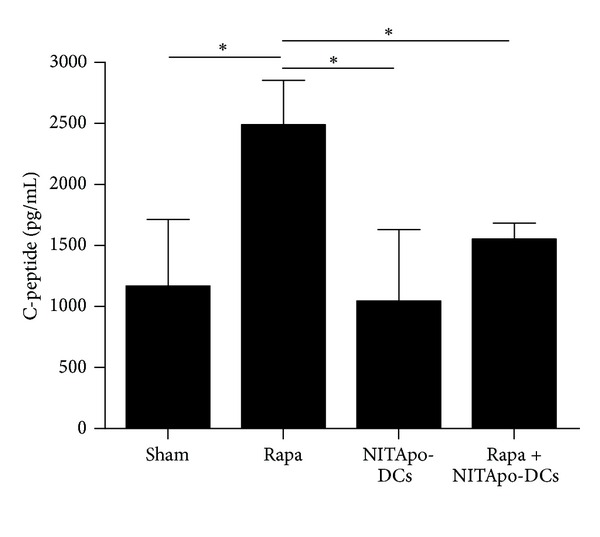
Endogenous insulin secretion ameliorates in mice treated with rapamycin but the effect is counteracted when combined with tolerogenic DCs. Serum C-peptide levels determined by ELISA at the end of the follow-up (30 days). Data from 3–6 animals/group are expressed as means ± SD. *Means significant differences, *P* < 0.05.

Serum C-peptide levels reflected pancreatic insulin contents. To determine the recovery of *β*-cell function, C-peptide concentrations in sera were determined at the end of the study. No significant differences were observed when compared mice treated with tolerogenic DCs to mice from the control sham group. Treatment with rapamycin increases insulin secretion in NOD mice: C-peptide levels were significantly higher in mice treated with rapamycin when compared to those in the sham group, *P* < 0.05. However, C-peptide levels in mice treated with tolerogenic DCs or simultaneously treated with tolerogenic DCs-based immunotherapy and rapamycin were similar to those in the sham group (Figure 3) and significantly lower than those in mice treated with rapamycin (*P* < 0.05).

## 4. Discussion

The NOD mouse model has proven to be an important tool for the study of new therapeutic targets and preclinical studies in T1D. More than 200 immune interventions have been described to prevent diabetes in NOD mice [8, 9] and a few have restored insulin secretion.

In a previous work we demonstrated that immature DCs pulsed with apoptotic islet cells prevent T1D in NOD, correlating with significant reductions of insulitis, costimulatory signals and the secretion of proinflammatory cytokines [2]. T1D prevention was achieved using DCs loaded with apoptotic islet cells, but not with apoptotic bodies from other cell type or unloaded DCs, thus demonstrating the antigenic specificity of immunotherapy. 

Therefore, the aim of this study was to reverse diabetes in diabetic mice by administering this immunotherapy. Since the focus of the study was to reverse diabetes and no effects were observed using tolerogenic DCs, we included a group of mice simultaneously treated with tolerogenic DCs and rapamycin. The lack of effect of the tolerogenic DCs-based immunotherapy in ameliorating endogenous insulin secretion, even when an immunosuppressant was administered to diabetic mice, could be due to several factors. On the one hand, the stage of insulitis at the time of immunotherapy administration was very severe, thus hindering the recovery of beta cell mass. On the other hand, the design of the immunotherapy (which was optimal for the prevention of the disease) could not have been appropriate for diabetes reversal. The amount of DCs and the number of doses could be too low for recovering endogenous insulin secretion. 

Surprisingly, rapamycin does not improve the effects of immunotherapy in NOD mice. Immunosuppressants have been reported to impair human *β*-cell function and survival [10] and for that reason these agents have not any effect in ameliorating insulin secretion. This is in contrast to data in prediabetic NOD mice wherein rapamycin significantly protected animals from disease development but did not reverse the course of the disease after T1D onset [11, 12]. However, rapamycin combined with other experimental immunotherapies that successfully prevent and cure T1D (e.g., anti-CD3- antibodies) [13] exerts a detrimental effect on disease outcome for as long as it is administered [14].

Our results showed a significant decrease in glycaemia in mice treated with tolerogenic DCs when compared to mice treated with rapamycin alone or in combination with tolerogenic DCs. However, all diabetic mice were hyperglycaemic during the follow-up and daily injections of insulin were required for survival. Although the differences are not significant, the lower insulitis score values achieved in our study correspond to mice treated with rapamycin alone or in combination with tolerogenic DCs-based immunotherapy. This drug seems to have an effect in a tendency to decrease insulitis, as expected for an immunosuppressor agent. We also observed that endogenous insulin secretion ameliorates in mice treated with rapamycin. However, when combined with tolerogenic DCs, the effect of rapamycin is counteracted, thus indicating that the combination of immunosuppressants with tolerogenic DCs does not increase the ability of tolerogenic DCs to recover insulin secretion in NOD mice.

## 5. Conclusion

In conclusion, immunotherapy with tolerogenic DCs alone or in combination with the immunosuppressant rapamycin has no effects on restoring insulin secretion in diabetic NOD mice. We believe that immunotherapy for curing T1D must be a combination of agents, dampening inflammation, re-establishing peripheral immunological tolerance, and helping islet regeneration to recover *β*-cell mass.
